# Invasive Pneumococcal Disease after Routine Pneumococcal Conjugate Vaccination in Children, England and Wales

**DOI:** 10.3201/eid1901.120741

**Published:** 2013-01

**Authors:** Shamez N. Ladhani, Mary P.E. Slack, Nick J. Andrews, Pauline A. Waight, Ray Borrow, Elizabeth Miller

**Affiliations:** Author affiliations: Health Protection Services Colindale, London, UK (S.N. Ladhani, M.P.E. Slack, N.J Andrews, P.A Waight, E. Miller);; Health Protection Agency, Manchester, UK (R. Borrow)

**Keywords:** Invasive pneumococcal disease, risk factors, serotypes, meningitis, outcome, vaccination, bacteria, England, Wales, children

## Abstract

We assessed known risk factors, clinical presentation, and outcome of invasive pneumococcal disease (IPD) in children 3–59 months of age after introduction of the 7-valent pneumococcal conjugate vaccine (PCV7) in England and Wales. During September 2006–March 2010, a total of 1,342 IPD episodes occurred in 1,332 children; 14.9% (198/1,332) had comorbidities. Compared with IPD caused by PCV7 serotypes (44/248; 17.7%), comorbidities were less common for the extra 3 serotypes in the 10-valent vaccine (15/299; 5.0%) but similar to the 3 additional PCV13 serotypes (45/336; 13.4%) and increased for the 11 extra serotypes in 23-valent polysaccharide vaccine (PPV23) (39/186; 21.0%) and non-PPV23 serotypes (38/138; 27.5%). Fifty-two (3.9%) cases resulted from PCV7 failure; 9 (0.7%) case-patients had recurrent IPD. Case-fatality rate was 4.4% (58/1,332) but higher for meningitis (11.0%) and children with comorbidities (9.1%). Thus, comorbidities were more prevalent in children with IPD caused by non-PCV13 serotypes and were associated with increased case fatality.

## Abstract 2

Nonvaccine serotypes occur more often among children with comorbid conditions.

In September 2006, the United Kingdom introduced the 7-valent pneumococcal conjugate vaccine (PCV7) into the national childhood immunization program for receipt at 2, 4, and 13 months of age (*1*). At the same time, a 12-month catch-up campaign was initiated that offered 2 vaccine doses to 2–8-month-old infants and 1 dose to 12–24-month-old children (*1*). The program rapidly achieved high vaccine coverage (*2*) and was highly effective (*3*), resulting in a rapid reduction in invasive pneumococcal disease (IPD) caused by the serotypes in PCV7 (PCV7-IPD; serotypes 4, 6B, 9V, 14, 18C, 19F, and 23F), particularly in children <2 years of age, for whom PCV7-IPD decreased by 98% by 2009–10 (*4*). Because conjugate vaccines induce high antibody levels that reduce carriage in vaccinated children and, therefore, transmission of *Streptococcus pneumoniae* to others, PCV7-IPD also declined by >75% in older age groups through indirect protection (herd immunity) (*4*). Moreover, although this reduction was offset by an increase in IPD caused by serotypes not included in PCV7 (serotype replacement disease), IPD decreased 34% overall across all age groups during 2009–10 (56% in children <2 years of age) (*4*). The introduction of a 13-valent vaccine (PCV13) in April 2010 provided protection against 2 of the key replacing serotypes, 7F and 19A (*5*); however, concern remains about the potential for further replacement disease with non-PCV13 serotypes.

After PCV7 introduction, the Health Protection Agency (HPA) collected detailed clinical information about all laboratory-confirmed IPD cases in children eligible for PCV7 in England and Wales. To predict the potential long-term effect of higher-valent vaccines on childhood IPD, an understanding is needed of the characteristics of children in whom IPD developed and of the infecting serotypes during the PCV7 period. We describe the distribution of known risk factors, clinical features, and outcome of illness in children with IPD in the cohort eligible for PCV7 in England and Wales.

## Methods

### IPD Surveillance

The HPA conducts enhanced surveillance for IPD in England and Wales (*4*). The HPA routinely collects computerized hospital laboratory reports of invasive pneumococcal isolates and actively requests referral of isolates to its national Reference Laboratory for serotyping. The laboratory reports are regularly reconciled with serotype data into a single dataset. We report on all IPD case-patients 3–59 months of age in the birth cohorts eligible for PCV7 (children born since September 2004) in whom IPD was diagnosed during the 43-month period spanning September 4, 2006–March 31, 2010 (before PCV13 introduction). The HPA has approval under Patient Information Advisory Group Section 60 of the Health and Social Care Act 2001 to process confidential patient information for public health purposes (www.legislation.hmso.gov.uk/si/si2002/20021438.htm).

In children with >1 IPD episode, only the first episode was included in the analysis except where repeat IPD episodes were described. Vaccination status was obtained by telephone from the child’s general practitioner, followed by a questionnaire to the general practitioner and/or pediatrician requesting clinical information, including known risk factors for IPD (*6*). Incomplete questionnaires and questionnaires that were not returned after a reminder letter was sent were followed up by telephone.

### Definitions

An IPD case was defined as culture of *S. pneumoniae* from a normally sterile site or, for culture-negative cases, detection of pneumococcal DNA in cerebrospinal or pleural fluid. Meningitis was defined as *S. pneumoniae* identified in cerebrospinal fluid through culture and/or PCR or clinical and/or radiologic features of meningitis with *S. pneumoniae* isolated from blood culture. Lower respiratory tract infection (LRTI) was defined as *S. pneumoniae* in empyema fluid or in blood with radiologic and/or clinical diagnosis of pneumonia. Septicemia was defined as *S. pneumoniae* cultured in blood with no distinctive clinical syndrome. Repeat samples from sterile sites within 30 days from the same person were regarded as part of the same episode.

PCV7 vaccine failure was defined as PCV7-IPD occurring at least 14 days after 2 doses in children <12 months of age or after 1 dose in children >12 months of age (irrespective of the number of previous PCV7 doses). The 3 extra serotypes in 10-valent PCV (PCV10) were 1, 5, and 7F; the additional 3 PCV13 serotypes were 3, 6A, and 19A; and the additional 11 serotypes in the 23-valent polysaccharide vaccine (PPV23) were 2, 8, 9N, 10A, 11A, 12F, 15B, 17F, 20, 22F, and 33F. Fatal cases were followed up by requesting a hospital discharge summary and a postmortem report where appropriate. Death caused by IPD was defined as *S. pneumoniae* identified from a normally sterile site 1) before death, with clinical, laboratory, and/or radiologic evidence of invasive bacterial infection; or 2) at postmortem examination, with histopathologic evidence of invasive bacterial infection and/or a report by the histopathologist that the pathogen contributed to the death.

### Data Analysis

Data were exported to Stata version 11.0 (StataCorp, College Station, TX, USA) for analysis. Mid-year population estimates were obtained from the Office for National Statistics (www.statistics.gov.uk). Continuous variables that did not follow a normal distribution were described as median and interquartile ranges (IQR) and compared by using the Mann-Whitney U test. Proportions were compared by using the χ^2^ test or Fisher exact test, as appropriate.

Multivariable logistic regression was used to calculate the adjusted odds ratio (aOR) and 95% CIs for 1) comorbidity with increasing age in years at disease onset, after adjustment for time since PCV7 introduction and sex; 2) comorbidity with vaccination status among PCV7-IPD cases after adjustment for age and time since PCV7 introduction; and 3) specific clinical features (e.g., meningitis, LRTI) as binary outcome variables and infecting pneumococcal serotype groups as explanatory variables after adjustment for sex, comorbidities, time since PCV7 introduction, and vaccination status. We also used a multivariable logistic regression model to evaluate risk factors for death; explanatory variables were age and time since PCV7 introduction as continuous variables and sex, vaccination status, infecting serotype group, comorbidities, and clinical features as categorical variables. Multinomial logistic regression was used to study the association between comorbidities and serotype groups after adjustment for age, sex, vaccination status, and time since PCV7 introduction.

## Results

During September 2006–March 2010, a total of 1,342 IPD cases occurred in 1,332 children 3–59 months of age. Median age at disease onset was 14.5 months (IQR 9.0–26.6 months);198 (14.9%) of the 1,332 patients had underlying comorbidity (Table 1), which, after adjustment for study year and sex, increased with age (aOR 1.17, 95% CI 1.04–1.33, p = 0.013). Malignancy/immunosuppression (56 cases) accounted for approximately one fourth of comorbidities, followed closely by congenital heart disease (36 cases), given that an additional 17/23 (74.9%) children with Down syndrome also had congenital heart disease (Table 2). Septicemia was the main clinical feature, followed by meningitis and LRTI (Table 1). Clinical presentation with meningitis decreased with age, whereas LRTI increased and accounted for more than one third of cases among children 2–5 years of age (Table 1). Comorbidities were present in 8.0% (19/237) of children with LRTI, 11.3% (34/300) with meningitis, 19.9% (154/772) with septicemia, and 17.4% (4/23) with other conditions.

**Table 1:** Characteristics of children with IPD in the cohort eligible for PCV7, England and Wales, September 4, 2006–March 31, 2010*

Characteristic	Age group, mo, no. (%)			Total, n = 1,332
	3–11, n = 507	12–23, n = 446	24–59, n = 379	
Male sex	307 (60.6)	253 (56.7)	220 (58.0)	780 (58.6)
No comorbidity	441 (87.0)	392 (87.9)	301 (79.4)	1,134 (85.1)
Any comorbidity	66 (13.0)	54 (12.1)	78 (20.6)	198 (14.9)
Serotype group				
PCV7	95 (18.7)	106 (23.8)	47 (12.4)	248 (18.6)
Extra 3 PCV10	86 (17.0)	78 (17.5)	135 (35.6)	299 (22.4)
Extra 3 PCV13	134 (26.4)	112 (25.1)	90 (23.7)	336 (25.2)
Extra 11 PPV23	82 (16.2)	62 (13.9)	42 (11.1)	186 (14.0)
Other	55 (10.8)	39 (8.7)	44 (11.6)	138 (10.4)
Not known	55 (10.8)	49 (11.0)	21 (5.5)	125 (9.4)
PCV7-IPD cases				
Year 1	66/142 (46.5)	88/136 (64.7)	15/25 (60.0)	169/303 (55.8)
Year 2	17/104 (16.3)	13/90 (14.4)	13/69 (18.8)	43/263 (16.3)
Year 3	11/114 (9.6)	4/116 (3.4)	7/134 (5.2)	22/364 (6.0)
Year 4 (36–43 mo)	1/92 (1.1)	1/55 (1.8)	12/130 (9.2)	14/277 (5.1)
Comorbidity				
Prematurity	58 (11.4)	44 (9.9)	28 (7.4)	130 (9.8)
Septicemia	272 (53.6)	291 (65.2)	209 (55.1)	772 (58.0)
Meningitis	195 (38.4)	70 (15.7)	35 (9.2)	300 (22.5)
LRTI	23 (4.5)	82 (18.4)	132 (34.8)	237 (17.8)
Other	17 (3.4)	3 (0.7)	3 (0.8)	23 (1.7)
>1 PCV7 vaccine dose	410 (80.9)	315 (70.6)	309 (81.5)	1,034 (77.6)
Hemolytic uremic syndrome†	8 (1.6)	11 (2.5)	5 (1.3)	24 (1.8)
Antimicrobial resistance				
Penicillin‡	4/191 (2.1)	5/154 (3.2)	4/118 (3.4)	13/465 (2.8)
Erythromycin	9/153 (5.9)	16/129 (12.4)	4/92 (4.3)	29/374 (7.8)
Died and had	27/507 (5.3)	16/446 (3.6)	15/379 (4.0)	58/1,332 (4.4)
No comorbidity	17/437 (3.9)	13/389 (3.3)	10/295 (3.4)	40/1,121 (3.6)
Comorbidity	10/70 (14.3)	3/57 (5.3)	5/84 (6.0)	18/211 (8.5)
Septicemia	7/272 (2.6)	5/291 (1.7)	6/209 (2.9)	18/772 (2.3)
Meningitis	18/195 (9.2)	9/70 (12.9)	6/35 (17.1)	33/300 (11.0)
LRTI	1/23 (4.3)	2/82 (2.4)	3/132 (2.3)	6/237 (2.5)
Other	1/17 (5.9)	0/3 (0)	0/3 (0)	1/23 (4.3)
Serotype group				
PCV7	3/95 (3.2)	6/106 (5.7)	2/47 (4.3)	11/248 (4.4)
PCV10	3/86 (3.5)	0/78 (0)	2/135 (1.5)	5/299 (1.7)
PCV13	8/134 (6.0)	3/112 (2.7)	5/90 5.6)	16/336 (4.8)
PPV23	2/82 (2.4)	5/62 (8.1)	2/42 (4.8)	9/186 (4.8)
Other	7/55 (12.7)	0/39 (0)	3/44 (6.8)	10/138 (7.2)

*IPD, invasive pneumococcal disease; PCV7, 7-valent pneumococcal conjugate vaccine, PCV10, 10-valent pneumococcal conjugate vaccine; PCV13, 13-valent pneumococcal conjugate vaccine; PPV23, 23-valent polysaccharide vaccine; PCV7-IPD cases, IPD caused by the serotypes in PCV7; LRTI, lower respiratory tract infection. †The responsible serotypes were 19A (13 cases, 52%), 7F (4 cases, 16%), 3 (3 cases, 12%), 1 (2 cases, 8%), 11A (1 case, 4%), and 22A (1 case, 4%). ‡Two isolates from each age group were of intermediate penicillin resistance.

**Table 2:** Comorbidities in children with invasive pneumococcal disease, England and Wales, September 4, 2006–March 31, 2010

Comorbidity	Age group, mo, no. (%)			Total, n = 1,332
	3–11, n = 507	12–23, n = 446	24–59, n = 379	
Total	66 (13.0)	54 (12.1)	78 (20.6)	198 (14.9)
Malignancy/immunosuppression	6 (9.1)	12 (22.2)	38 (48.7)	56 (28.3)
Hematologic malignancy	0	1	23	24
Solid-organ malignancy	3	6	7	16
Transplant*	1	1	3	5
Primary immunodeficiency	2	4	3	9
HIV infection	0	0	2	2
Congenital heart disease	22 (33.3)	7 (13.0)	7 (9.0)	36 (18.2)
Down syndrome†	9 (13.6)	5 (9.3)	9 (11.5)	23 (11.6)
Respiratory disease	10 (15.2)	11 (20.4)	6 (7.7)	27 (13.6)
Chronic lung disease	5	5	4	14
Congenital anomaly	5	5	1	11
Severe asthma on oral steroids	0	1	1	2
Gastrointestinal disease	7 (10.6)	4 (7.4)	2 (2.6)	13 (6.6)
Biliary atresia	5	0	1	6
Long-term total parenteral nutrition dependency‡	2	4	1	7
Neurologic disease	7 (10.6)	7 (13.0)	7 (9.0)	21 (10.6)
Cerebral insult/palsy	4	1	4	9
Congenital anomaly	2	4	2	8
Cochlear implant	0	1	1	2
Ventricular-peritoneal shunt	1	1	0	2
Sickle cell disease	2 (3.0)	3 (5.6)	6 (7.7)	11 (5.6)
Renal disease	3 (4.5)	5 (9.3)	3 (3.8)	11 (5.6)
Congenital anomaly	3	1	1	5
Metabolic renal condition	0	1	0	1
Nephrotic syndrome	0	2	2	4
Chronic renal failure	0	1	0	1

*Transplant of the heart (2), liver (1), bone marrow (1), and kidney (1), †Congenital heart disease also was reported in 6/9, 4/5, and 7/9 children with Down syndrome in the 3 age groups, respectively. ‡Four with postnecrotizing enterocolitis with surgical resection of bowel, 2 with protein-losing enteropathy, and 1 with malabsorption syndrome.

### Pneumococcal Serotypes causing IPD

Serotype information was available for 90.6% (1,207/1,332) of first episodes (Technical Appendix Table 1). The proportion of PCV7-IPD cases decreased with time since PCV7 introduction in all age groups (Figure; Table 1). During the last 7 months of surveillance, the extra 6 serotypes in PCV13 accounted for 69.0% (191/277) of IPD cases for which the organism was serotyped.

**Figure:**
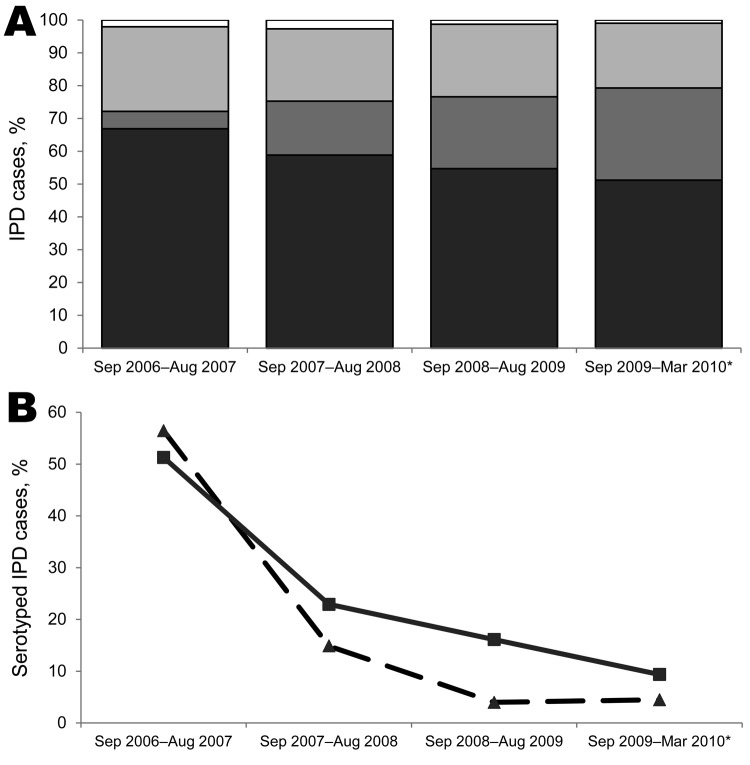
IPD clinical cases in PCV7-eligible children since PCV7 introduction, England and Wales, September 4, 2006–March 31, 2010. A) Distribution of cases. Black bar sections, bacteremia; dark gray bar sections, lower respiratory tract infection; light gray bar sections, meningitis; white bar sections, other. B) Proportion of serotyped cases caused by PCV7 serotypes in healthy children (dashed line) and children with comorbidities (solid line). The prevalences of comorbidity among IPD cases during the 4 time periods were 13,5%, 17.7%, 17.0%, and 10.7%, and the case-fatality rates were 5.6%, 4.3%, 4.3%, and 3.0%, respectively. *Data included for 7 months only. IPD, invasive pneumococcal disease; PCV7, 7-valent pneumococcal conjugate vaccine.

### Comorbidities

The proportion of children with comorbidities did not alter with time since PCV7 introduction (Figure). Among PCV7-IPD cases, although a higher proportion of children with comorbidities had been previously vaccinated with >1 PCV7 dose (22/44 [50.0%] vs. 69/204 [33.8%]), the difference was not significant after adjustment for age at disease onset and time since PCV7 introduction (aOR 1.58, 95% CI 0.72–3.47, p = 0.25). However, the proportion with comorbidity differed by serotype group (p<0.001) (Table 3). The difference was due to a low proportion with comorbidity for the extra 3 PCV10 serotypes and high proportion for the additional 11 PPV23 serotypes, as well as non-PPV23 serotypes. This relationship remained significant after adjustment for age, vaccination status, and time since PCV7 introduction by using multinomial logistic regression where serotype group was set as the outcome variable.

**Table 3:** Association between infecting pneumococcal serotype group and presence of comorbidity, England and Wales, September 4, 2006–March 31, 2010*

Serotype group	Comorbidity, no./total (%)	aOR† (95% CI)	p value
All PCV7-IPD cases‡	44/248 (17.7)		
3 extra PCV10 serotypes (1, 5, 7F)	15/299 (5.0) §	0.24 (0.13–0.45) against PCV7	<0.001
3 extra PCV13 serotypes (3, 6A, 19A)	45/336 (13.4)§	0.72 (0.46–1.13) against PCV7	0.15
		3.58 (1.93–6.66) against PCV10‡	<0.001
11 extra PPV23 serotypes	39/186 (21.0)	1.23 (0.76–1.99) against PCV7	0.40
		6.02 (3.16–11.5) against PCV10	<0.001
		1.68 (1.04–2.71) against PCV13	0.033
Remaining non-PPV23 serotypes	38/138 (27.5)	1.76 (1.07–2.89) against PCV7	0.025
		8.57 (4.46–16.5) against PCV10	<0.001
		2.39 (1.46–3.93) against PCV13	0.001
		1.42 (0.85–2.40) against PPV23	0.18

*aOR, adjusted odds ration; PCV7, 7-valent pneumococcal conjugate vaccine; PCV10, 10-valent pneumococcal conjugate vaccine; PCV13, 13-valent pneumococcal conjugate vaccine; PPV23, 23-valent polysaccharide vaccine. †Odds ratio adjusted for age, vaccination status, and time since PCV7 introduction by using a multinomial logistic regression with serotype group as the outcome. ‡Serotypes 4, 6B, 9V, 14, 18C, 19F, and 23F. §Comorbidity was lower for each of the 3 extra PCV10 serotypes, 1 (7/129, 5.4%), 5 (1/17, 5.9%), and 7F (7/153, 4.6%), compared with the additional 3 serotypes in PCV13, 3 (13/98, 13.3%), 6A (12/47, 25.5%), and 19A (20/191, 10.5%).

### Clinical Features

After PCV7 introduction, the proportion of children with LRTI increased, even after adjustment for age at disease onset, sex, comorbidities, and infecting serotype group (aOR 1.19/year, 95% CI 1.01–1.41, p = 0.045). When analyzed by serotype group, after adjustment for sex, comorbidities, age, time since PCV7 introduction, and vaccination status, the 3 extra PCV10 serotypes were less likely to cause meningitis (aOR 0.43, 95% CI 0.26–0.74, p = 0.002) and more likely to cause LRTI (aOR 6.49, 95% CI 2.73–15.5, p<0.001) than were PCV7 serotypes. The observation was the same for the 3 additional PCV13 serotypes (aOR 0.46, 95% CI 0.28–0.74, p = 0.001, and aOR 11.2, 95% CI 4.77–26.3, p<0.001, respectively), and the findings remained significant even after the 3 additional PCV13 serotypes were replaced with serotype 3 only in the logistic regression model (aOR for meningitis 0.16, 95% CI 0.06–0.43, p<0.001; aOR for LRTI 37.0, 95% CI 14.4–95.3, p<0.001). The prevalence of comorbidity in children with serotype 3 IPD was similar to that in children with IPD caused by serotypes 6A and 19A (13/98 [13.3%] vs. 32/238 [13.4%], p = 0.97) although the median age at disease onset was higher (22.6 months [IQR 14.6–36.1 months] vs. 12.1 months [8.6–19.3 months], p<0.001).

### Antimicrobial Susceptibility

Results of penicillin susceptibility testing were reported for 465 isolates; 13 (2.8%) exhibited intermediate (6 [1.3%]) or complete (7 [1.5%]) resistance and belonged to serotypes 19A (4 isolates), 19F, 1, 15A, and 9V (1 isolate each) among serotyped isolates (Table 1). Only 1 child with penicillin-resistant IPD had a comorbidity (malignancy), and another had recently returned from southern Europe, but all survived. Results for erythromycin susceptibility testing were reported for 374 isolates, of which 29 (7.8%) were resistant, mainly among serotypes 14 (13 isolates) and 19F (4 isolates). Four isolates were reported as resistant to penicillin and erythromycin. None of these children had comorbidity; 3 developed septicemia, 1 had meningitis, and none died. Penicillin resistance remained stable during the surveillance period, whereas erythromycin resistance declined from 19.4% (21/108) in the first year after vaccine introduction to 1.4% (1/73) in the final year, mainly because of declines in serotypes 14 and 19F.

### Vaccine Failure

PCV7-IPD occurred in 248 (20.5%) of 1,207 serotyped cases, and 52 (3.9%) of 1,332 children with IPD had 53 episodes of PCV7 vaccine failure, including 1 fully vaccinated cochlear implant recipient with 2 distinct meningitis episodes 10 months apart. Serotypes 6B (18/53 cases, 34.0%) and 19F (16/53, 30.2%) were responsible for almost two thirds of PCV7 vaccine failures. Case-patients with PCV7 vaccine failure were more likely to have comorbidities (15/52 [28.8%] vs. 166/1,155 [14.4%] case-patients with known serotype, p = 0.004). Only 1 case-patient with PCV7 vaccine failure, who had immune deficiency, died of pneumococcal meningitis 2 months after receiving a PCV7 catch-up dose in the second year of life.

The additional 11 PPV23 serotypes were responsible for 14% of serotyped IPD cases overall and 22% for those with comorbidity. Of the 78 children >2 years of age who had comorbidities and would have been eligible for PPV23, IPD caused by the 11 additional PPV23 serotypes developed in 17 (21.8%). Only 4 (5.1%) of the 78 children had received PPV23 before their IPD episode; IPD caused by a PPV23 serotype developed in 2 of these, 1 with sickle cell disease who had been fully vaccinated with PCV7 and PPV23 and in whom serotype 19A IPD subsequently developed and 1 with severe developmental delay who had received 1 catch-up dose of PCV7 and PPV23 but in whom serotype 6B IPD subsequently developed. The other 2 PPV23-vaccinated children, both with sickle cell disease and fully vaccinated with PCV7 and PPV23, developed serotypes 6A and 23A IPD, respectively.

### Repeat IPD Episodes

Nine (0.7%) children had repeat IPD episodes. Eight had 2 episodes each, and 1 had 3 episodes (Technical Appendix Table 2). Four had known comorbidities (44.4% vs. 14.7% [194/1,323] for the rest of the cohort; p = 0.012), 1 was a PCV7 vaccine failure, and none died.

### Case-Fatality Rates

Sixty-two children died, including 4 with multiple comorbidities who died several weeks after recovering from IPD; their deaths were attributed to complications of chronic liver disease (2 children), *Escherichia coli* septicemia (1 child), and group A streptococcal septicemia (1 child). The IPD-attributable case-fatality rate (CFR) was, therefore, 4.4% (58/1,332); almost one third (18/58 [31.0%]) of children who died had comorbidities. Seven (12.1% [1 with comorbidity]) died at home; 8 (13.8% [6 with comorbidities]) died on the way to the hospital; 12 (20.7% [6 with comorbidities]) died in the emergency department; and 31 (53.5% [5 with comorbidities]) died in the intensive care unit. One toddler, in whom *Staphylococcus aureus* meningitis had developed at 7 months of age, died of pneumococcal meningitis at 13 months of age, and IRAK-4 mutation was subsequently diagnosed at postmortem examination.

Of the 51 deaths in children for whom serotype was known, CFR for PCV7-IPD cases was equally distributed among the 7 serotypes, and 10/11 (90.9%) deaths occurred after 4 months of age, of which 9 were in unvaccinated children and, therefore, potentially vaccine-preventable. CFR did not vary with time since PCV7 introduction (Figure) and was lower for IPD caused by the extra 3 PCV10 serotypes (1.7%) but not statistically significant (Table 1) and. CFR for serotype 3 IPD (5/98 cases, 5.1%) was similar to the overall IPD CFR in this age group. After adjustment for age, sex, vaccination status, and time since PCV7 introduction in a logistic regression model, infecting serotype group was not associated with death. On the other hand, meningitis (aOR 6.43, 95% CI 3.36–12.3, p<0.001) and presence of comorbidities (aOR 2.70, 95% CI 1.35–5.38, p = 0.005) were significantly associated with death.

## Discussion

Comorbidities were identified in 15% of PCV7-eligible children in England and Wales, with malignancy/immunosuppression and congenital heart disease each accounting for one quarter of reported comorbidities. Compared with PCV7-IPD cases, the prevalence of comorbidity was significantly lower for IPD cases caused by the 3 additional PCV10 serotypes and higher for non-PCV13 IPD cases. Overall CFR was low and independently associated with meningitis and comorbidity but not with infecting serotype group.

The comorbidities in the cohort reported here are typical of children considered at higher risk for IPD (*6*–*9*). Other population-based studies that had differing definitions for comorbidities and age ranges have reported comorbidities in 10%–36% of children with IPD (*7*,*10*–*13*). In Massachusetts, USA, 16% of 578 children <18 years of age who had IPD during 2001–2007 had comorbidity (mainly immunosuppression) (*9*). The US Active Bacterial Core surveillance program identified comorbidity in only 3% of childhood IPD cases before PCV7 introduction in 1998–99, but this proportion increased to 7% in 2006–07 (p = 0.003), with similar increases in adults and the elderly; this finding suggests that the replacing serotypes after the decline in PCV7-IPD might be less virulent and thus more likely to infect vulnerable children with comorbidities (*14*). The lower prevalence of comorbidities in the Active Bacterial Core surveillance might be explained partly by inclusion of children who had blood cultures performed in an outpatient setting, whereas blood cultures in the United Kingdom are almost always taken in the hospital and, therefore, capture more severe IPD cases (*4*). Moreover, changes in clinical practice after PCV7 introduction, such as fewer blood cultures from previously healthy children with fever seeking outpatient care, might have contributed to the increased comorbidity prevalence between the 2 periods in the United States (*14*).

In the cohort reported here, the 3 extra PCV10 serotypes were more likely to affect healthy children. Serotypes 1, 5, and 7 (which are included in both PCV10 and PCV13) are known to be highly invasive and mainly affect previously healthy persons but appear to cause less severe disease, as determined by various clinical severity scores and requirement for intensive care, and have a lower CFR (*15*). In children, a meta-analysis of 7 different datasets found that serotypes 1, 5, and 7 were infrequently isolated among carriage strains but had the highest potential for invasive disease, whereas serotypes that were more likely to be carried (e.g., 6B, 19F, and 23F) were less likely to be invasive (*16*).

A higher prevalence of comorbidity was identified for IPD cases caused by the 11 additional PPV23 serotypes as well as non-PPV23 IPD cases, suggesting that the remaining serotypes after PCV13 introduction in the United Kingdom might be less virulent. In England and Wales, PPV23 uptake for high-risk persons is low, particularly for children (*17*), and is consistent with the low PPV23 vaccination rates observed among children with comorbidities in the IPD cohort reported here. That such a low proportion of high-risk children were vaccinated with the nationally recommended PPV23 and 2 of the 4 PPV23-vaccinated case-patients had IPD resulting from 1 of the PPV23 serotypes is concerning and merits further investigation on the use of polysaccharide vaccines in high-risk persons.

In keeping with other studies, PCV7 vaccine failure (3.9%) was uncommon (*18*–*20*), and nearly one third of case-patients had comorbidities. A recent study in the United States reported 4% of 753 IPD cases diagnosed during a 27-month period as PCV7 vaccine failures, with 37% of the case-patients with vaccine failure having comorbidities (*20*). In that study, PCV7-vaccinated children with comorbidities were almost 3× more likely to develop PCV7-IPD than were vaccinated children without comorbidities, even after we controlled for various confounders (*20*). In another case–control study in the United States that involved 782 children 3–59 months of age who had IPD, vaccine effectiveness of >1 PCV7 dose against PCV7-IPD was 96% (95% CI 93%–98%) for healthy children and 81% (95% CI 57%–92%) for children with comorbidities (*21*). Similar estimates of 97% (95% CI 92%–98%) and 88% (95% CI 78%–94%), respectively, were obtained by using the indirect cohort (Broome) method to measure direct protection afforded by PCV7 (*22*). In the United Kingdom, where a 12-month catch-up campaign offering PCV7 to all children <2 years of age was introduced at the same time as routine infant vaccination, vaccine effectiveness estimated by using the indirect cohort method was similar for children with comorbidities and healthy children receiving the nationally recommended immunization courses, albeit with wide confidence intervals for the different comorbidities and immunization schedules (*3*).

In our study and the US studies, serotypes 6B and 19F were predominantly responsible for PCV7 vaccine failures. Similarly, recurrent IPD was reported in only 9 children (0.7%) and, like vaccine failure, was more likely to occur in children with comorbidities. The relatively long duration between episodes and the different infecting serotypes suggest re-infection rather than persistent infection as a consequence of, for example, inadequate or inappropriate therapy. Before routine pneumococcal vaccination, a US population-based surveillance study reporting 318 cases of recurrent IPD identified persons with HIV and children <5 years of age with chronic illness as the 2 main risk groups (*23*). A more recent US study reported recurrent IPD in 90 of 4,067 children with IPD over 12 years, with comorbidities identified in >80% (*24*). Recurrent IPD cases declined significantly after PCV7 introduction, although age at re-infection or proportion with comorbidities during the pre- and post-PCV7 periods did not differ (*24*). Despite the smaller age range and shorter follow-up period, our study supports the low risk for recurrent IPD after PCV7 introduction, even though we are unable to compare with pre-PCV7 rates. Although 5 of 9 children with recurrent IPD had no reported comorbidities, some could have undetected immunologic abnormalities, as was identified in a child with fatal IPD who had IRAK-4 deficiency, for example (*25**–**27*). Children in whom IPD developed, particularly because of vaccine failure, should be carefully assessed, and children who have a history of >1 serious invasive infection should be investigated for possible immune deficiency along with their close family members because further infections possibly could be prevented through appropriate immunization and/or antimicrobial prophylaxis.

The extensive follow-up of IPD cases, particularly the fatal cases, helped us to more accurately estimate CFR and assess IPD-associated deaths. Overall, IPD-associated CFR was low and independently associated with comorbidity and meningitis. The finding that meningitis was diagnosed in one quarter of mainly healthy children is concerning given the significantly higher CFR and association with the most severe long-term neurodevelopmental complications (reported in >30% of survivors) among pathogens causing bacterial meningitis in children (*28*).

Like all large-scale epidemiologic studies, our study has limitations. Not all hospital laboratories in England and Wales routinely report or submit clinical isolates to the HPA. However, by combining multiple data sources and actively requesting submission of invasive pneumococcal isolates to the HPA national Reference Laboratory for confirmation and serotyping, we believe that our surveillance captures most laboratory-confirmed IPD cases and, because the same surveillance method has been in place since PCV7 introduction, enables comparison of trends over time. The study is also limited by the relatively short follow-up period for identifying vaccine failure cases and recurrent episodes. However, given the significant decline in carriage of PCV7 serotypes (*29*), we are unlikely to see many cases of PCV7-IPD, particularly in young children where vaccine coverage remains high (*30*). The changing pattern of IPD after PCV7 introduction emphasizes the need for continued epidemiologic and molecular surveillance across all age groups. In addition to assessing the role of PPV23 in protecting children with comorbidities, further studies are required to develop new strategies and vaccines with broader coverage to prevent IPD in children who are most susceptible and reduce deaths associated with pneumococcal meningitis.

Technical Appendix Pneumococcal serotype distribution in children with invasive pneumococcal disease over time since introduction of 7-valent pneumococcal conjugate vaccine and children with >1 invasive pneumococcal disease episode. England and Wales, September 4, 2006–March 31, 2010.
