# Supplementary material for: Invasive Pneumococcal Disease after Routine Pneumococcal Conjugate Vaccination in Children, England and Wales
Source: Emerg Infect Dis. 2013 Jan;19(1):61–8. doi: 10.3201/eid1901.120741 (PMC3557991; doi:10.3201/eid1901.120741)
Supplement: Technical Appendix — Pneumococcal serotype distribution in children with invasive pneumococcal disease over time since introduction of 7-valent pneumococcal conjugate vaccine and children with >1 invasive pneumococcal disease episode. England and Wales, September 4, 2006–March 31, 2010. [file 12-0741-Techapp-s1.pdf]

Article DOI: http://dx.doi.org/10.3201/eid1901.120741

Invasive Pneumococcal Disease after Routine Pneumococcal Conjugate Vaccination in Children, England and Wales

Technical Appendix

Technical Appendix Table 1. Pneumococcal serotype distribution in children with invasive pneumococcal disease over time since PCV7 introduction, England and Wales, September 4, 2006–March 31, 2010*

	Time period, no. cases (%)				
Serotype	Sep 2006–Aug 2007	Sep 2007–Aug 2008	Sep 2008–Aug 2009	Sep 2009–April 2010†	All cases
PCV7	169 (49.6)	43 (14.4)	22 (5.6)	14 (4.7)	248 (18.6)
14	62 (18.2)	7 (2.3)	3 (0.8)	0 (0.0)	72 (5.4)
6B	39 (11.4)	18 (6.0)	6 (1.5)	3 (1.0)	66 (5.0)
19F	22 (6.5)	9 (3.0)	5 (1.3)	8 (2.7)	44 (3.3)
23F	18 (5.3)	4 (1.3)	4 (1.0)	1 (0.3)	27 (2.0)
18C	17 (5.0)	0 (0.0)	0 (0.0)	1 (0.3)	18 (1.4)
9V	9 (2.6)	4 (1.3)	1 (0.3)	1 (0.3)	15 (1.1)
4	2 (0.6)	1 (0.3)	3 (0.8)	0 (0.0)	6 (0.5)
PCV10	26 (7.6)	68 (22.7)	105 (26.7)	100 (33.4)	299 (22.4)
7F	16 (4.7)	25 (8.4)	56 (14.2)	56 (18.7)	153 (11.5)
1	9 (2.6)	32 (10.7)	45 (11.5)	43 (14.4)	129 (9.7)
5	1 (0.3)	11 (3.7)	4 (1.0)	1 (0.3)	17 (1.3)
PCV13	56 (16.4)	73 (24.4)	116 (29.5)	91 (30.4)	336 (25.2)
19A	27 (7.9)	33 (11.0)	62 (15.8)	69 (23.1)	191 (14.3)
3	8 (2.3)	26 (8.7)	44 (11.2)	20 (6.7)	98 (7.4)
6A	21 (6.2)	14 (4.7)	10 (2.5)	2 (0.7)	47 (3.5)
Remaining	52 (15.2)	79 (26.4)	121 (30.8)	72 (24.1)	324 (24.3)
22F	6 (1.8)	7 (2.3)	23 (5.9)	16 (5.4)	52 (3.9)
33F	5 (1.5)	17 (5.7)	15 (3.8)	6 (2.0)	43 (3.2)
15C	4 (1.2)	10 (3.3)	14 (3.6)	4 (1.3)	32 (2.4)
7	0 (0.0)	6 (2.0)	6 (1.5)	8 (2.7)	20 (1.5)
12F	6 (1.8)	1 (0.3)	10 (2.5)	2 (0.7)	19 (1.4)
8	5 (1.5)	4 (1.3)	4 (1.0)	4 (1.3)	17 (1.3)
15B	3 (0.9)	3 (1.0)	6 (1.5)	4 (1.3)	16 (1.2)
38	2 (0.6)	8 (2.7)	4 (1.0)	2 (0.7)	16 (1.2)
10A	5 (1.5)	2 (0.7)	7 (1.8)	1 (0.3)	15 (1.1)
6C	4 (1.2)	0 (0.0)	5 (1.3)	6 (2.0)	15 (1.1)
Other	12 (3.5)	21 (7.0)	27 (6.9)	19 (6.4)	79 (5.9)
Not known	38 (11.1)	36 (12.0)	29 (7.4)	22 (7.4)	125 (9.4)

*PCV7, 7-valent pneumococcal conjugate vaccine, PCV10, 10-valent pneumococcal conjugate vaccine; PCV13, 13-valent pneumococcal conjugate vaccine; PPV23, 23-valent polysaccharide vaccine.
†Includes data for 7 months only.

Technical Appendix Table 2. Children with >1 invasive pneumococcal disease episode, England and Wales, September 4, 2006–March 31, 2010*

Sex	Age at disease, mo.	Co-morbidity	Diagnosis	Serotype
F†	30	Cochlear implant	Meningitis	4
	41		Meningitis	4
F	25	Immune deficiency	Septicemia	14
	40	Born prematurely at 30 wks' gestation	Septicemia	15B
F	10	Down syndrome	Septicemia	Unknown
	18		Septicemia	7
F	30	Malignancy	Septicemia	6A
	37		Septicemia	7F
M	6	None	Septicemia	3
	27		Lower respiratory tract infection	1
M	14	None	Lower respiratory tract infection	Unknown
	23		Lower respiratory tract infection	Unknown
F	5	None	Septicemia	Unknown
	30		Septicemia	24F
F	19	None	Septicemia meningitis	6B
	25			6B
F	13	None	Septicemia	18C
	15		Septicemia	18B
	39		Meningitis	22F

*None of these children died.
†Vaccine failure case.
